# Ameliorating effect of *Celastrus paniculatus* standardized extract and its fractions on 3-nitropropionic acid induced neuronal damage in rats: possible antioxidant mechanism

**DOI:** 10.1080/13880209.2017.1285945

**Published:** 2017-02-05

**Authors:** Jai Malik, Maninder Karan, Rachna Dogra

**Affiliations:** University Institute of Pharmaceutical Sciences – Centre of Advanced Study, Panjab University, Chandigarh, India

**Keywords:** 3-Nitropropionic acid, Huntington's disease, Jyotishmati, Malkangni

## Abstract

**Context:***Celastrus paniculatus* Wild. (Celasteraceae) (CP) is a well-known Ayurvedic ‘Medhya Rasayana’ (nervine tonic), used extensively as a neuro-protective and memory enhancer, and in different central nervous system disorders.

**Objective:** To evaluate the effect of CP against 3-nitropropionic acid (3-NP) induced Huntington's disease (HD) like symptoms in Wistar male rats.

**Materials and methods:** The ethanol extract of CP seeds (CPEE), prepared by maceration, was standardized on the basis of linoleic acid content (6.42%) using thin layer chromatography densitometric analysis. Protective effect of CPEE (100 and 200 mg/kg) and its various fractions, viz., petroleum ether (40 mg/kg), ethyl acetate (2.5 mg/kg), *n*-butanol (7 mg/kg) and aqueous (18 mg/kg), administered orally for 20 days, against 3-NP (10 mg/kg, i.p. for 14 days) was assessed by their effect on body weight, locomotor activity, grip strength, gait pattern and cognitive dysfunction and biochemical parameters for oxidative damage in the striatum and cortex regions of the brain.

**Results:** CPEE (100 and 200 mg/kg) treated animals exhibited a significant (*p* < 0.05) improvement in behavioural and oxidative stress parameters in comparison to only 3-NP treated animals. Amongst various tested fractions of CPEE, aqueous fraction (AF) at 18 mg/kg exhibited maximum reversal of 3-NP induced behavioural and biochemical alterations, and was therefore also tested at 9 and 36 mg/kg. CPEE (100 mg/kg) and AF (36 mg/kg) exhibited maximum and significant (*p* < 0.05) attenuation of 3-NP induced alterations in comparison to 3-NP treated rats.

**Conclusions:** CPEE has a protective action against 3-NP induced HD like symptoms due to its strong antioxidant effect.

## Introduction

Huntington’s disease (HD) is an autosomal dominant neurodegenerative disorder characterized by progressive motor dysfunction including chorea, dyskinesia, dystonia, deterioration of memory, weight loss and other CNS disorders like anxiety, depression, etc. (Frank 2014). Altered genetic transcription, oxidative stress, glutamate excitotoxicity and mitochondrial dysfunction are considered as the mainstay in the pathophysiology of the disorder that lead to the neuronal damage mainly in the striatal and cortex regions of the brain (Krobitsch & Kazantsev 2011).

Various chemical and genetic experimental models are being used to uncover the pathological mechanisms and to formulate an effective therapeutic intervention for HD. 3-Nitropropionic acid (3-NP), a chemical agent that produces HD like symptoms in rodents, is widely used to evaluate the efficacy of various test compounds against HD. 3-NP is a mycotoxin produced by various members of the genus *Astragalus* Linn. (Leguminosae) and *Arthrinium* Sacc. (Apiosporaceae) fungi that inactivates succinic dehydrogenase, a key enzyme in the tricarboxylic acid cycle (TCA) and oxidative phosphorylation (complex II) reactions, under *in vivo* conditions (Tunez et al. 2010; Shinomol & Muralidhara 2011). 3-NP administration also increases the concentration of free fatty acids and free radicals [reactive oxygen species (ROS)] in the brain, resulting in elevated oxidative stress (Shinomol & Muralidhara 2011) leading to HD like symptoms.

*Celastrus paniculatus* Willd. (Celastraceae) (CP), commonly known as Jyotishmati, Malkangni and Kangani has been categorized as a *Medhya Rasayana* (nervine tonic) in Ayurveda (Nadkarni 2002). Mainly its seeds and seed oil are used for its memory enhancing and neuroprotective properties (Bhanumathy et al. 2010a). Various sesquiterpenoid polyalcohols and esters (malkanguniol, malkangunin, polyalcohol A–D, celapnin); alkaloids (paniculatine, celastrine); phenolic triterpenoids (celastrol, paniculatadiol); fatty acids (oleic, linoleic, linolenic, palmitic, stearic and lignoceric acid) and agarofuran derivatives have been isolated from the seeds and seed oil of CP (Hertog et al. 1973; Wagner et al. 1974; Tu et al. 1991; Tu & Chen 1993; Bhanumathy et al. 2010a). Ample studies have exhibited the memory enhancing (Nalini et al. 1995; Gattu et al. 1997; Lekha et al. 2010; Bhanumathy et al. 2010b) and neuroprotective (Godkar et al. 2003, 2004, 2006) potential of the seeds and seed oil of CP. The plant has also exhibited antianxiety (Rajkumar et al. 2007), antispermatogenic (Bidwai et al. 1990), anti-inflammatory and analgesic activities (Ahmad et al. 1994). A significant antioxidant activity of the plant has been suggested as the major mechanism for its memory enhancing and neuroprotective activities (Kumar & Gupta 2002).

Since, antioxidants play a sizeable role in controlling HD like symptoms (Johri & Beal 2012; Gil-Mohapel et al. 2014) and CP has exhibited neuroprotective activities by virtue of its antioxidant effect, the present study was designed to evaluate the effect of standardized extract of CP seeds and its fractions on 3-NP induced HD like symptoms.

## Materials and methods

### Plant material

Seeds of CP were procured from a local market in Chandigarh in the month of September 2016 and were authenticated by Dr. Sunita Garg, Chief Scientist, National Institute of Science Communication and Information Resources (NISCAIR), New Delhi, vide certificate no. NISCAIR/RHMD/Consult/2014/2529/108, dated 09 October 2014.

### Chemicals and solvents

The solvents used for extraction and analytical procedures were of LR and AR grade, respectively. Standard linoleic acid and 3-NP were procured from Sigma-Aldrich (St. Louis, MO). Pre-coated silica gel 60 F-254 TLC aluminium plates (20 × 20 cm, 0.2 mm thick) were purchased from E. Merck, Darmstadt, Germany. All the chemicals used for biochemical estimation were procured from Central Drug House (CDH), New Delhi, India. Distilled water was used wherever mentioned.

### Instruments

A complete HPTLC system (CAMAG, Muttenz, Switzerland) equipped with an Automated TLC sampler (ATS-4), TLC scanner 3, AMD and winCATS integration software (version 4.01) was used for sample analysis. Twin trough glass development chamber was used for TLC development. Tissue homogenizer, cooling centrifuge (REMI Instruments, Mumbai, India) and UV spectrophotometer (Perkin Elmer, Singapore) were used for biochemical estimations.

### Experimental animals

Male Wistar rats (220–250 g) obtained from Central Animal House of Panjab University Chandigarh, Punjab, India, were used for the project work. The experimental protocol was approved by the Institutional Animal Ethics Committee (IAEC). Animals were kept in polyacrylic cages and maintained under standard housing conditions with 12 h light/dark reverse cycle. The food in the form of dry pellets and water were made available *ad libitum*. All behavioural experiments were carried out between 9:00 and 17:00 h.

### Extraction of CP seeds and fractionation of the extract

Coarsely powdered seeds (750 g) were extracted for 72 h by maceration using ethanol. After every 24 h, the used ethanol was replaced with fresh ethanol. Occasional shaking was done during the extraction period. The ethanol extract of seeds (CPEE) was filtered, pooled and concentrated under vacuum using rotary evaporator. The CPEE (180 g, 24%w/w) thus obtained, was used for biological activity (50 g) and fractionation (100 g). Fractionation was done by suspending the extract in water (500 mL) followed by sequential partitioning with different solvents (5 × 250 mL), viz., petroleum ether, ethyl acetate and *n*-butanol, in increasing order of polarity.

### Standardization of CPEE using TLC densitometry

CPEE was standardized by determining the linoleic acid content through TLC densitometric analysis using HPTLC system (CAMAG, Muttenz, Switzerland). All the sample and standard applications were done in triplicate.

#### Preparation of stock solution of standard

Linoleic acid (10 mg) was weighed accurately and dissolved in 10 mL of methanol to obtain a concentration of 1 mg/mL. The stock solution was further diluted with methanol to obtain the final concentration of 0.1 mg/mL.

#### Preparation of calibration curve for linoleic acid

Standard stock solution of linoleic acid was diluted appropriately to get solutions of different concentration, viz., 20, 40, 60, 80 and 100 ng. Each concentration (10 μL) was applied in triplicate on the TLC plate. The plate was developed using chromatographic conditions as described above. The area under curve (AUC) was recorded for each peak and graph was plotted using mean AUC against the corresponding concentrations.

#### Preparation of test solution

The shade dried coarsely powdered seeds of CP (5 g) were macerated with 50 mL ethanol for 72 h as described earlier. The extract was filtered through Whatman fine paper, concentrated under vacuum and the final volume was made up to 50 mL with ethanol. The sample stock solution was appropriately diluted, and 10 μL of the final solution was applied on TLC plate followed by development and scanning.

#### Sample application

The samples were applied in triplicate in the form of bands (width 8 mm) with ATS-4 (sample applicator) on pre-coated TLC plate. The distance between tracks was kept 10 mm and an application rate of 80 nL/s was employed for applying bands. Linear ascending development was carried out in a twin trough glass chamber saturated with mobile phase. Densitometric scanning was performed using TLC scanner 3 in the absorbance/reflectance mode.

#### Chromatographic conditions for estimation of linoleic acid

Linoleic acid was estimated in the test sample (plant extract) by using a validated HPTLC method (Tandon & Sharma 2011). Briefly, after application of test sample, the plates were developed using mobile phase, petroleum ether:diethyl ether:glacial acetic acid (9:3:0.1), in a pre-saturated (5 min) twin trough glass chamber. During post development process, the plates were dried in a current of hot air, derivatized using anisaldehyde–sulphuric acid and were scanned at 400 nm using TLC scanner 3.

### Dose preparation and treatment schedule

3-Nitropropionic acid was dissolved in normal saline (pH 7.4) and administered at a dose of 10 mg/kg, i.p. to animals for 14 days. CPEE was evaluated against 3-NP induced neurotoxicity at two oral doses of 100 and 200 mg/kg. The doses were selected on the basis of study done previously (Kumar & Gupta 2002). The fractions were evaluated at a dose (calculated on the basis of the yield of each fraction) equivalent to the dose of alcoholic extract that exhibited maximum activity. The fraction(s) that showed maximum effect was further evaluated at a lower and higher dose. The doses of CPEE (100 and 200 mg/kg) and its fractions, viz. petroleum ether (PF, 40 mg/kg), ethyl acetate (EF, 2.5 mg/kg), *n*-butanol (BF, 7 mg/kg) and aqueous (AF, 9, 18 and 36 mg/kg) were prepared by suspending in 0.1% sodium carboxy-methyl-cellulose (CMC) solution, and were administered by oral route (p.o.) at a constant volume of 0.5 mL per 100 g of body weight. Animals were randomly divided into 10 groups consisting of eight animals each. Group 1 was the vehicle-treated group; Group 2 animals received 3-NP (10 mg/kg) for 14 days, Groups 3 and 4 received CPEE (100 and 200 mg/kg) + 3-NP (10 mg/kg), respectively; Groups 5 received PF (40 mg/kg) + 3-NP (10 mg/kg); Group 6 received EF (2.5 mg/kg) + 3-NP (10 mg/kg); Group 7 received BF (7 mg/kg) + 3-NP (10 mg/kg); Groups 8–10 received AF (9, 18 and 36 mg/kg) + 3-NP (10 mg/kg), respectively. The complete protocol was designed for 20 days (designated as day −5 to day 15). The control group was administered vehicle for all the 20 days. For first six days (designated as day −5 to day 0) only extract/fractions were administered at different dose levels to the animals of different test groups (Malik et al. 2015). On day 1 (six days after extract/fraction treatment), 3-NP was administered to the animals of each test group 1 h after extract/fraction administration. The same treatment schedule was followed for the next 14 days.

### Pharmacological studies

#### Body weight

Animal body weight was recorded on the first and last day of the study and the change in body weight (%) was calculated.

#### Grip strength

Assessment of grip strength was done to determine muscle tone, balance and motor co-ordination in the animals. It provides an easy way to test the effects of drugs, lesions in brain, aging, brain damage or diseases on motor coordination or fatigue resistance in rodents. Grip strength task was evaluated by using rotarod apparatus on day 1, 5, 10 and 15 after 3-NP injection. Before actual recording on rotarod apparatus, the animals were given a prior training session for acclimatization. During the rotarod test, rats were placed on the rotating rod at a speed of 25 rpm, and fall off time was recorded with an upper ceiling of 300 s (Kumar & Kumar 2009a; Malik et al. 2015).

#### Gait abnormality

Gait abnormality indicates the motor in-coordination particularly of the hind limbs. It was assessed by narrow beam walk apparatus. This test has been very effective in detecting motor capabilities in rats. The apparatus consists of 50 cm wooden strip supported by two pedestals at each end, with height of 100 cm above the ground and the rats traverse the narrow beam suspended between a start platform and their home cage. A prior training, to walk over a beam, was given to rats for five days before testing. A ceiling of 120 s was employed at the end of which the animal was removed and placed in the cage by hand. Time taken by the animal to traverse from the start platform to their home cage along with the number of slips was recorded (Kumar & Kumar 2009b; Malik et al. 2015).

#### Locomotor activity

Effect on locomotor activity was assessed by using an actophotometer as it provides task-specific practice of standing and walking by optimizing the sensory cues to generate improved motor activity for mobility, standing and walking after neurologic injury. The effect on locomotor activity was assessed on day 1, 5, 10 and 15. The motor activity was detected by infrared beams above the floor of the testing area. Animals were placed individually in the activity chamber for 3 min as a habituation period before performing actual motor activity tasks. Each animal was observed over a period of 10 min and activity was expressed as counts per 10 min (Kumar & Kumar 2009b; Malik et al. 2015).

#### Memory impairment

The effect on learning and retention was examined by the performance of rats in Morris water maze. The animals were trained to swim to a platform in a circular pool located in a test room. The pool was filled with water and was placed in a large room where several brightly coloured cues, external to the maze, were visible and could be used by the rats for spatial orientation. A movable circular platform, 9 cm in diameter, mounted on a column, was placed in the pool 1 cm below the water level for the maze acquisition test. The position of the cues remained unchanged throughout the study. The platform was fixed in the centre of one of the four quadrants and remained in that location for the duration of the experiment. The rats were exposed to four consecutive daily training trials starting from the first day of 3-NP administration, with each trial having a ceiling time of 120 s, and a trial interval of approximately 5 min. For each trial, each rat was put into the water at one of four starting positions, the sequence of which being selected randomly. During the test trials, the rats were placed in the tank at the same starting point, with their heads facing the wall, and were allowed to swim before climbing onto the platform. After climbing onto the platform, the animal was allowed to remain there for 20 s, before it was returned to its cage. The escape platform was kept in the same position relative to the distal cues. If the rat failed to reach the escape platform within the allowed time, it was gently placed on the platform and allowed to remain there for the same amount of time. The time to reach the platform was recorded as transfer latency (TL), and was measured on day 5, 10 and 15. On the next day, i.e., 24 h after the last trial, the platform was removed and the animal was randomly released at any one of the edges (North, South, East and West) facing the wall of the pool and tested for the retention of the memory. The time spent by the animal in the target quadrant (where platform was placed) was recorded (Kumar & Kumar 2009b; Malik et al. 2015).

### Biochemical estimations

#### Dissection and homogenization

After behavioural assessments, animals were killed by decapitation immediately for biochemical analysis. The brains were removed, cortex and striatum were separated by putting on ice. A 10% (w/v) tissue homogenate was prepared in 0.1 M phosphate buffer (pH 7.4). The homogenate was centrifuged at 10,000×*g* for 15 min. Aliquots of supernatant was separated and used for biochemical estimations.

#### Various biochemical parameters

The effect of CPEE and its various fractions against 3-NP induced neurotoxicity was also evaluated by measuring their effect on different biochemical parameters, viz., malondialdehyde (Wills 1966), nitrite (Green et al. 1982) and reduced glutathione (Ellman 1959) levels, and catalase (Luck 1971) and superoxide dismutase activity (Kono 1978). The total protein content was measured in all brain samples by the Biuret method using bovine serum albumin (BSA) as a standard (Gornall et al. 1949).

### Statistical analysis

The results were expressed as mean ± S.D. The data of behavioural studies were statistically analysed using two-way analysis of variance (ANOVA) followed by Bonferroni’s *post hoc* test. The data of body weight, retention test in MWM and biochemical estimations were analysed by one-way ANOVA followed by Tukey’s *post hoc* test. *p* < 0.05 was considered statistically significant.

## Results

### Extraction of the plant material, fractionation and standardization

**Figure 1.**
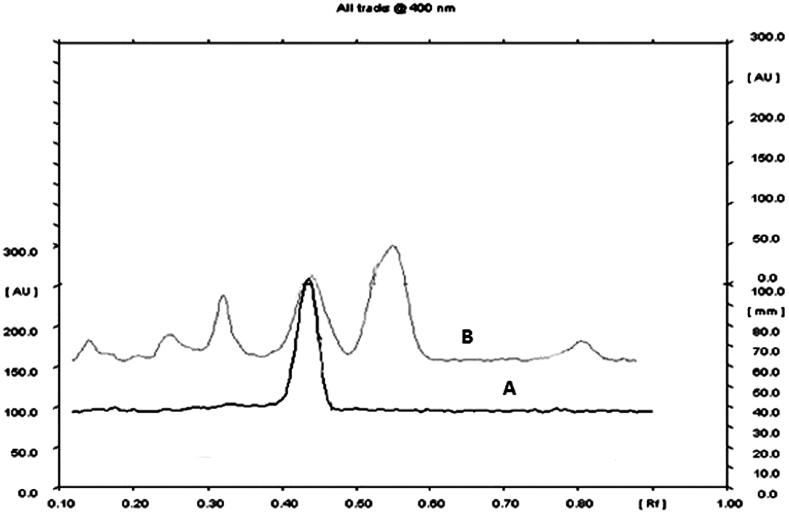
TLC densitometric chromatogram for standard linoleic acid (A) and CPEE (B).

Alcoholic extraction of the coarsely powdered seeds (750 g) of CP by maceration (72 h) yielded 180 g of the CPEE. One portion of the CPEE (100 g) was kept for fractionation and other portion (50 g) was stored at −20 °C for biological activity. CPEE was fractionated using various solvents in increasing order of polarity to get their respective fractions, viz., PF (55 g), EF (3.7 g), BF (13.8 g) and AF (27 g). CPEE was standardized on the basis of linoleic acid content using TLC densitometric analysis (Figure 1). The linoleic acid content was found to be 6.42% w/w in the seeds of *C. paniculatus*.

### Effect of CPEE and its various fractions on body weight in the 3-NP treated rats

**Figure 2.**
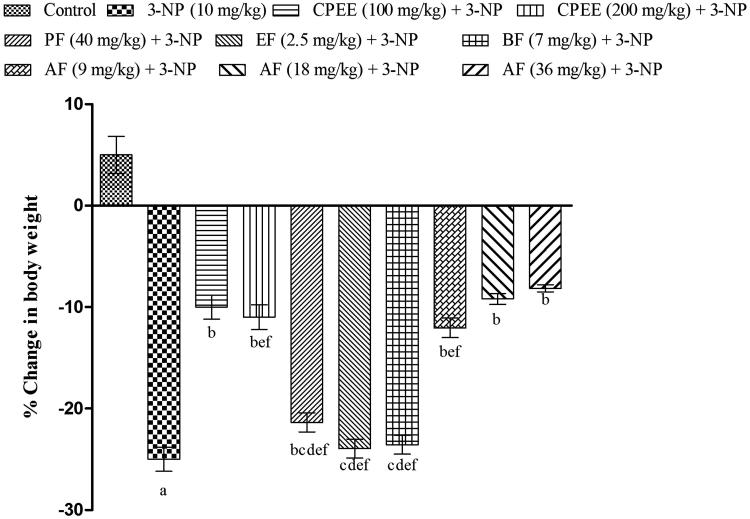
Effect of CPEE and its various fractions on the body weight of 3-NP treated rats. Results are expressed as mean (%) change in body weight ± SD (*n* = 8); ^a^*p* < 0.05 vs control; ^b^*p* < 0.05 vs 3-NP; ^c^*p* < 0.05 vs CPEE 100 mg/kg; ^d^*p* < 0.05 vs AF (9 mg/kg); ^e^*p* < 0.05 vs AF (18 mg/kg); ^f^*p* < 0.05 vs AF (36 mg/kg). Results are compared using one way analysis of variance followed by Tukey’s *post hoc* test. CPEE: ethanol extract of *Celastrus paniculatus* seeds; PF: petroleum ether fraction; EF: ethyl acetate fraction; BF: *n*-butanol fraction; AF: aqueous fraction.

There was a significant (*p* < 0.05) decrease in the body weight of the animals treated with 3-NP (10 mg/kg, i.p.) in comparison to vehicle-treated animals on the day 15 (Figure 2). Pretreatment with CPEE, PF and AF significantly attenuated the loss in body weight on 15th day in comparison to only 3-NP treated group (Figure 2). CPEE at both the dose levels exhibited significant protection against 3-NP induced decrease in body weight and the effect at both the dose levels was comparable to each other. Since AF (18 mg/kg) exhibited maximum activity amongst various fractions, it was further evaluated at a lower and a higher dose (9 and 36 mg/kg, respectively). AF attenuated the 3-NP induced decrease in body weight in comparison to control group with maximum activity at 36 mg/kg (though the effect was comparable (*p* > 0.05) to that of 18 mg/kg). Furthermore, CPEE (100 mg/kg) and AF (18 and 36 mg/kg) exhibited comparable effects. PF (40 mg/kg) also exhibited significant protection from 3-NP induced decrease in body weight, but the effect was significantly less than CPEE and AF.

### Effect of CPEE and its various fractions on grip strength in the 3-NP treated rats

**Figure 3.**
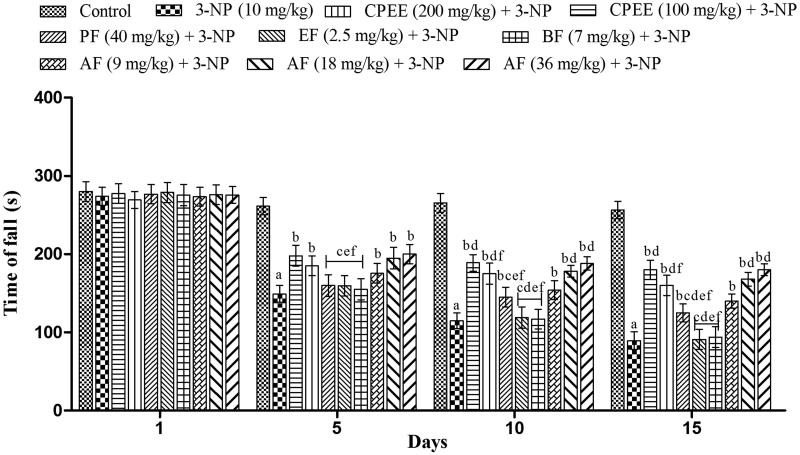
Effect of CPEE and its various fractions on the grip strength of 3-NP treated rats. Results are expressed as mean total time of fall ± SD (*n* = 8); ^a^*p* < 0.05 vs control; ^b^*p* < 0.05 vs 3-NP; ^c^*p* < 0.05 vs CPEE 100 mg/kg; ^d^*p* < 0.05 vs AF (9 mg/kg); ^e^*p* < 0.05 vs AF (18 mg/kg); ^f^*p* < 0.05 vs AF (36 mg/kg). Results are compared using two way analysis of variance followed by Bonferroni’s *post hoc* test. CPEE: ethanol extract of *Celastrus paniculatus* seeds; PF: petroleum ether fraction; EF: ethyl acetate fraction; BF: *n*-butanol fraction; AF: aqueous fraction.

3-NP significantly impaired grip strength of the animals on 5th, 10th and 15th day in comparison to vehicle treated animals in rotarod test (Figure 3). CPEE (100 and 200 mg/kg) pretreatment, significantly (*p* < 0.05) improved the 3-NP induced impairment in grip on day 5, 10 and 15 that was evident from the increase in time of fall of the animals. Pre-treatment with AF also exhibited a significant improvement in the grip strength on day 5, 10 and 15 in comparison to 3-NP treated animals (Figure 3). Apart from AF, PF (40 mg/kg) also exhibited significant improvement in grip strength but from day 10.

### Effect of CPEE and its various fractions on locomotor activity in 3-NP treated rats

**Figure 4.**
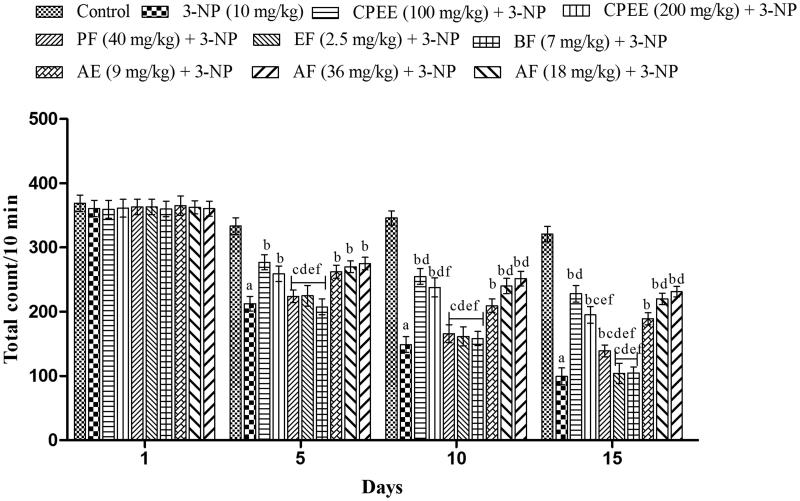
Effect of CPEE and its various fractions on locomotor activity of 3-NP treated rats. Results are expressed as mean total count ± SD (*n* = 8); ^a^*p* < 0.05 vs control; ^b^*p* < 0.05 vs 3-NP; ^c^*p* < 0.05 vs CPEE 100 mg/kg; ^d^*p* < 0.05 vs AF (9 mg/kg); ^e^*p* < 0.05 vs AF (18 mg/kg); ^f^*p* < 0.05 vs AF (36 mg/kg). Results are compared using two way analysis of variance followed by Bonferroni’s *post hoc* test. CPEE: ethanol extract of *Celastrus paniculatus* seeds; PF: petroleum ether fraction; EF: ethyl acetate fraction; BF: *n*-butanol fraction; AF: aqueous fraction.

As in the grip strength test, animals of each group exhibited a stable locomotor activity and showed no significant variation on day 1. CPEE (100 and 200 mg/kg) significantly (*p* < 0.05) abrogates the 3-NP induced decrease in the locomotor activity on day 5, 10 and 15 (Figure 4). CPEE (100 mg/kg) exhibited significantly (*p* < 0.05) better improvement in locomotor activity on day 10 and 15 in comparison to CPEE (200 mg/kg). Amongst various fractions of CPEE, AF (9, 18 and 36 mg/kg) showed a significant improvement in locomotor activity on day 5, 10 and 15 as compared with 3-NP treated group (Figure 4). PE (40 mg/kg) also exhibited significant improvement in locomotor activity but only on day 15.

### Effect of CPEE and its various fractions on the gait functions in 3-NP treated rats

**Figure 5.**
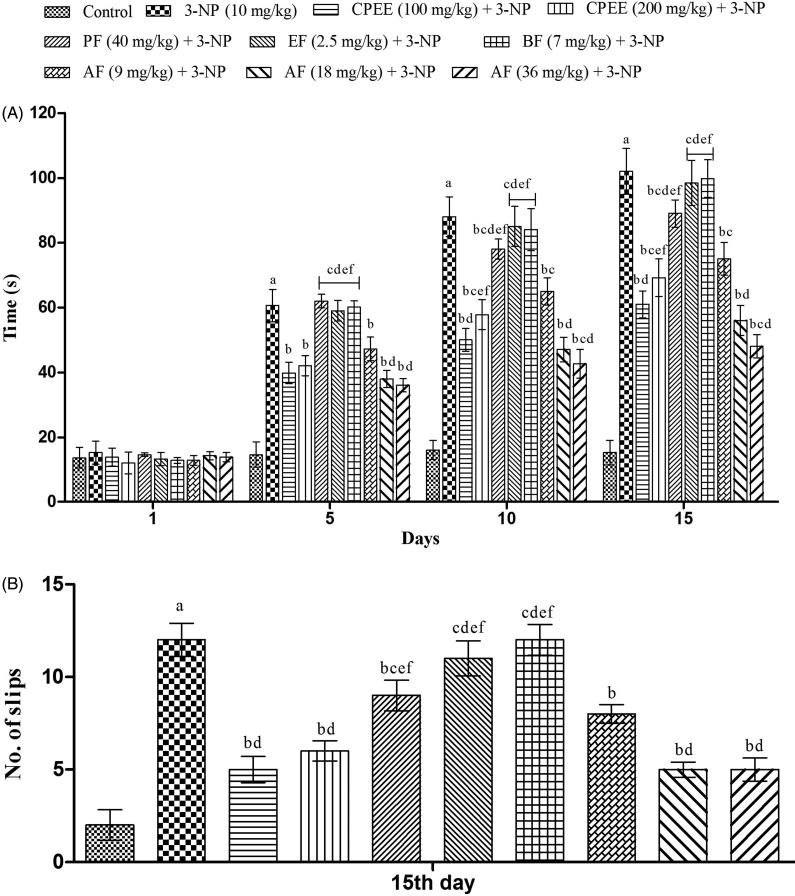
Effect of CPEE and its various fractions on gait functions of 3-NP treated rats. (A) Time taken to cross the narrow beam; (B) number of slips. Results are expressed as mean ± SD (*n* = 8); ^a^*p* < 0.05 vs control; ^b^*p* < 0.05 vs 3-NP; ^c^*p* < 0.05 vs CPEE 100 mg/kg; ^d^*p* < 0.05 vs AF (9 mg/kg); ^e^*p* < 0.05 vs AF (18 mg/kg); ^f^*p* < 0.05 vs AF (36 mg/kg). Results are compared using two way analysis of variance followed by Bonferroni’s *post hoc* test. CPEE: ethanol extract of *Celastrus paniculatus* seeds; PF: petroleum ether fraction; EF: ethyl acetate fraction; BF: *n*-butanol fraction; AF: aqueous fraction.

Systemic administration of 3-NP significantly increases the time taken by rats to traverse from start platform to their home cage as compared to the vehicle treated group on day 5, 10 and 15 (Figure 5(A)). CPEE (100 and 200 mg/kg) significantly reversed the 3-NP induced gait abnormalities of rats that was evident from the decrease in the time taken by them to cross the narrow beam and the number of slips (Figure 5(A,B)). AF pretreatment, at all the tested dose levels, significantly decreased the time taken by rats to cross the narrow beam and their number of slips on day 5, 10 and 15 in comparison to only 3-NP treated animals (Figure 5(A,B)). Furthermore, AF (36 mg/kg) exhibited maximum and significant improvement in the gait of rats when compared to CPEE (100 mg/kg) and AF (18 mg/kg) (Figure 5(A)). PF (40 mg/kg) also significantly improved the gait performance of rats on narrow beam in comparison to 3-NP treated rats from day 10 onwards.

### Effect of CPEE and its various fractions on learning and memory of 3-NP treated rats

**Figure 6.**
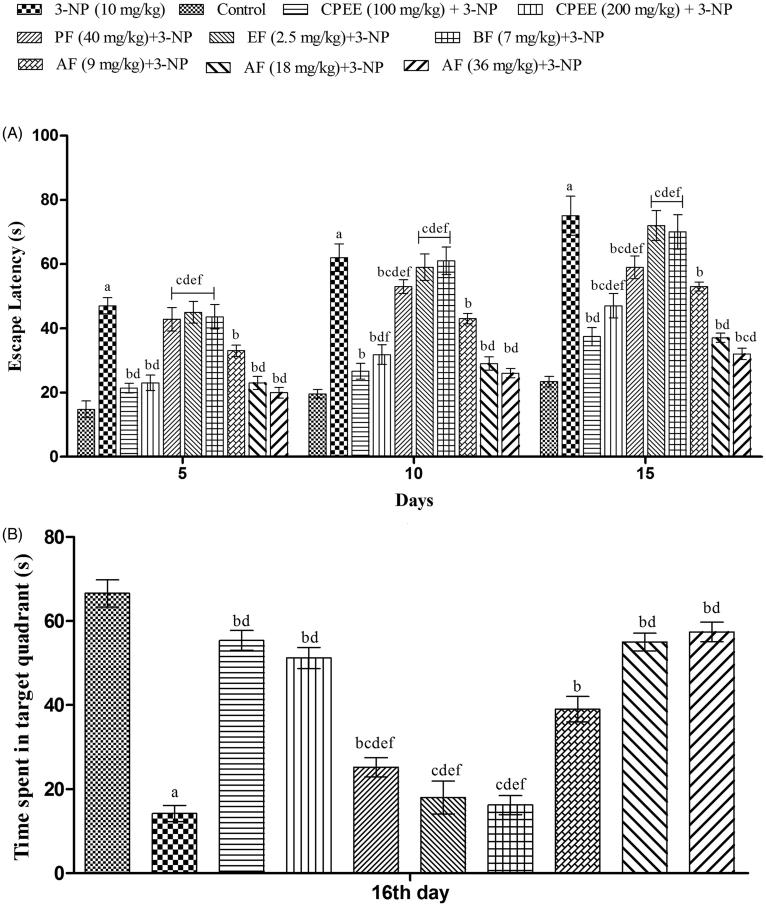
Effect of CPEE and its various fractions on memory functions of 3-NP treated rats on Morris water maze. (A) Time taken (s) by rats to reach platform (transfer latency); (B) time spent (s) in target quadrant. Results are expressed as mean ± SD (*n* = 8); ^a^*p* < 0.05 vs control; ^b^*p* < 0.05 vs 3-NP; ^c^*p* < 0.05 vs CPEE 100 mg/kg; ^d^*p* < 0.05 vs AF (9 mg/kg); ^e^*p* < 0.05 vs AF (18 mg/kg); ^f^*p* < 0.05 vs AF (36 mg/kg). Results are compared using two way analysis of variance followed by Bonferroni’s *post hoc* test. CPEE: ethanol extract of *Celastrus paniculatus* seeds; PF: petroleum ether fraction; EF: ethyl acetate fraction; BF: *n*-butanol fraction; AF: aqueous fraction.

The effect on learning was evaluated using Morris water maze test. Vehicle treated animals exhibited a significantly shorter escape latency in comparison to 3-NP treated animals after 5 day training, indicating learning impairment by 3-NP (Figure 6(A)). CPEE (100 and 200 mg/kg) treatments significantly (*p* < 0.05) improved memory performance, evident from shortened TL, on day 10 and 15 in comparison to 3-NP treated rats (Figure 6(A)).

AF (9, 18 and 36 mg/kg) treatment significantly (*p* < 0.05) attenuated the 3-NP induced learning impairment that was evident from the shortened mean TL on day 5, 10 and 15 in comparison to 3-NP treated rats (Figure 6(A)). PF (40 mg/kg) exhibited improvement in learning, but from day 10, in comparison to 3-NP treated rats.

On day 16, the platform was removed and the time spent by the rats in the target quadrant (where platform was placed) was recorded. More time spent in that quadrant by an animal indicates better remembrance of the platform position, and hence better memory. 3-NP treatment impaired the memory of rats which was evident from the shorter time spent by only 3-NP treated rats in target quadrant in comparison to vehicle treated rats (Figure 6(B)). However, CPEE (100 and 200 mg/kg) treatment significantly increased the time spent in target quadrant as compared to 3-NP treated animals (Figure 6(B)) thereby indicating improvement in memory consolidation of animals. As expected, AE (9, 18 and 36 mg/kg) treatment significantly increased the time spent in target quadrant as compared to 3-NP treated animals (Figure 6(B)).

### Effect of CPE and its various fractions on MDA, nitrite, catalase, SOD and GSH levels in striatum and cortex in brain of 3-NP treated rats

**Figure 7.**
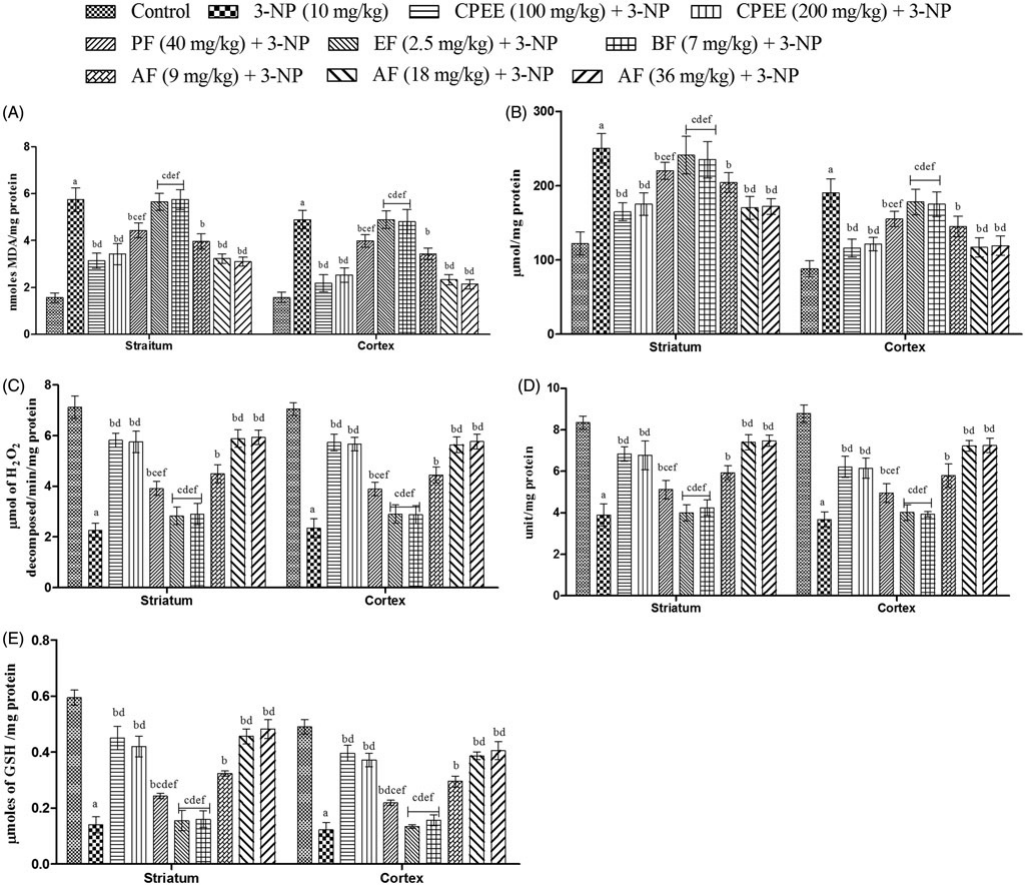
Effect of CPEE and its various fractions on oxidative parameters of 3-NP treated rats. (A) MDA levels; (B) nitrite levels; (C) catalase levels; (D) SOD levels; (E) reduced GSH levels. Results are expressed as mean ± SD (*n* = 8). ^a^*p* < 0.05 vs control; ^b^*p* < 0.05 vs 3-NP; ^c^*p* < 0.05 vs CPEE 100 mg/kg; ^d^*p* < 0.05 vs AF (9 mg/kg); ^e^*p* < 0.05 vs AF (18 mg/kg); ^f^*p* < 0.05 vs AF (36 mg/kg). Results are compared using one way analysis of variance followed by Tukey’s *post hoc* test. CPEE: Ethanol extract of *Celastrus paniculatus* seeds; PF: petroleum ether fraction; EF: ethyl acetate fraction; BF: *n*-butanol fraction; AF: aqueous fraction.

The results of behavioural studies were corroborated by the results of biochemical studies. There was a significant increase in MDA and nitrite levels in the striatum and cortex of the only 3-NP treated animals in comparison to the control group animals (Figure 7(A,B)). 3-NP treatment also decreased the levels of catalase, SOD and reduced GSH in comparison to vehicle treated group (Figure 7(C–E)). Pretreatment with CPEE (100 and 200 mg/kg) and AF (9, 18 and 36 mg/kg) significantly (*p* < 0.05) ameliorated the 3-NP induced alteration in MDA, nitrite, catalase, SOD and GSH levels in the striatum and cortex parts of the brain in comparison to only 3-NP treated animals. AF (18 and 36 mg/kg) exhibited comparable (*p* > 0.05) effects to that of CPEE (100 mg/kg).

## Discussion

The present study highlights the therapeutic potential of CPEE and its fractions, viz., PF, EF, BF and AF respectively, against 3-NP induced behavioural alterations and oxidative damage. CPEE (100 mg/kg), and amongst various fractions tested, AF (36 mg/kg), were most effective in ameliorating 3-NP induced behavioural alterations (improved body weight, locomotor, rotarod performance, balance beam walk performance and memory retention), and oxidative damage (attenuated lipid peroxidation, nitrite levels, restored catalase, SOD and GSH levels).

3-NP model is a widely used experimental model for evaluating the potential of various test substances that have propensity to develop into new therapeutic leads and/or agents against HD. It provides an advantage of producing striatal lesions rapidly after its administration. Further, 3-NP is a mitochondrial toxin that causes irreversible inhibition of SDH, a respiratory chain complex II enzyme, leading to body weight loss and motor abnormalities (reduced locomotor and grip strength, gait abnormalities, etc.) and increased oxidative stress. Supporting to the present investigation, HD patients have also exhibited dysphagia and loss of body weight (Djousse et al. 2002; Saydoff et al. 2003). Furthermore, it has been found that the body weight loss increases with the increase in the CAG repeats (Aziz et al. 2008). 3-NP induced loss in body weight could be partially due to factors outside the CNS, whereas alterations in locomotor and motor behaviour could be its specific action on striatum (basal ganglia) that control body movement (Saydoff et al. 2003). Dysfunction of cholinergic interneurons in striatal circuits or cell loss within the lateral striatum, ventral pallidum and entopeduncular nucleus has been found mainly responsible for abnormal behavioural symptoms in HD (Picconi et al. 2006). It shows that 3-NP produces alteration in motor behaviour by influencing striatum. However, the possibility of its effect on, and involvement of other brain areas such as cortex and hippocampus cannot be ignored (Silva et al. 2007). Pretreatment with CPEE and its AF fraction significantly restored the loss in body weight in 3-NP treated rats. It also attenuated other behavioural alterations following 3-NP administration. Previous reports have shown that substances with antioxidant property significantly restored the behavioural changes and oxidative defence level in 3-NP treated animals (Kumar et al. 2006; Kumar & Kumar 2010; Malik et al. 2015). *C. paniculatus* is well known for its neuroprotective effect by virtue of its antioxidant effect (Kumar & Gupta 2002; Badrul & Ekramul 2011) and this property could have been responsible for it protective effects in this study as well. 3-NP treatment significantly caused cognitive dysfunction that was evident from the increased TL, and decreased time spent in target quadrant. These findings are in agreement with earlier reports suggesting a variety of neurobehavioural abnormalities and cognitive deficit after 3-NP administration (Kumar & Kumar 2009b; Malik et al. 2015). It has also been reported that 3-NP produces lesion in hippocampal CA1 and CA3 pyramidal neurons, areas of the brain associated with cognitive task, leading to the impairment in cognitive functions (Sugino et al. 1999). Pretreatment with CPEE and AF exhibited significant improvement in the 3-NP induced cognitive dysfunctions. *C. paniculatus* is well known for its memory enhancing activity, and the results of the present study further strengthened the claim of *C. paniculatus* as potential memory enhancer.

Previous studies have shown that 3-NP significantly induced oxidative damage and impaired antioxidant defence enzymes in the brain (Perez-De La Cruz et al. 2009; Tunez & Santamaria 2009; Kumar & Kumar 2009b). The results of the present study are in concordance with those of the previous studies as 3-NP significantly induced oxidative damage (increased lipid peroxidation, nitrite concentration, and depleted catalase, SOD and reduced glutathione levels) in the striatum, and cortex regions of the rat brain. Antioxidants have shown significant neuroprotection against 3-NP induced neurotoxicity in various studies (Gopinath & Sudhandiran 2012; Chakraborty et al. 2014). Pretreatment with CPEE and its AF significantly restored the 3-NP induced oxidative alterations by virtue of their significant antioxidant activity. Furthermore, glutamate excitotoxicity also plays a major role in the pathophysiology of neurodegenerative disorders especially, HD (Andre et al. 2010), and glutamate antagonist helps in reversing some of the symptoms of HD (Mittal & Eddy 2013). Glutamate excitation, mainly the excitation of NMDA receptors, leads to high Ca^2+^ fluxes which further triggers a cascade of events including mitochondrial membrane depolarization, caspase activation, production of toxic oxygen and nitrogen free radicals, and cellular toxicity eventually leading to neuronal death (Dong et al. 2009). Glutamate receptor and/or NMDA receptor antagonist have shown a neuro-protective effect against such damage (Palmer 2001). *C. paniculatus* seeds and its aqueous extract have also shown neuroprotective effect against glutamate (Godkar et al. 2004) and hydrogen peroxide (Godkar et al. 2003) induced neurotoxicity. Aqueous extract of *C. paniculatus* have shown to antagonize both, NMDA receptors and its associated increase in Ca^2+^ fluxes, eventually protecting neurons from glutamate toxicity (Godkar et al. 2004).

Thus, in summary, the present study suggests the protective effect of *C. paniculatus* against 3-NP induced neurotoxicity could be due to its strong antioxidant effect and its role against glutamate toxicity by inhibiting NMDA receptors. However, more investigations are required to elucidate the cellular mechanisms of CP against 3-NP induced HD-like symptoms.

## References

[CIT0001] Ahmad F, Khan RA, Rasheed S. 1994 Preliminary screening of methanolic extracts of *Celastrus paniculatus* and *Tecomella undulata* for analgesic and anti-inflammatory activities. J Ethnopharmacol. 42:193–198. 7934089 10.1016/0378-8741(94)90085-x

[CIT0002] Andre VM, Cepeda C, Levine MS. 2010 Dopamine and glutamate in Huntington's disease: a balancing act. CNS Neurosci Ther. 16:163–178. 20406248 10.1111/j.1755-5949.2010.00134.x PMC3118459

[CIT0003] Aziz NA, Van Der Burg JMM, Landwehrmeyer GB, Brundin P, Stijnen T, Roos RAC, Group ES. 2008 Weight loss in Huntington disease increases with higher CAG repeat number. Neurology. 71:1506–1513. 18981372 10.1212/01.wnl.0000334276.09729.0e

[CIT0004] Badrul A, Ekramul H. 2011 Anti-Alzheimer and antioxidant activity of *Celastrus paniculatus* seed. Iran J Pharm Sci. 7:49–56.

[CIT0005] Bhanumathy M, Chandrasekar SB, Chandur U, Somasundaram T. 2010a Phyto-pharmacology of *Celastrus paniculatus*: an overview. Int J Pharm Sci Rev Res. 2:176–181.

[CIT0006] Bhanumathy M, Harish MS, Shivaprasad HN, Sushma G. 2010b Nootropic activity of *Celastrus paniculatus* seed. Pharm Biol. 48:324–327. 20645820 10.3109/13880200903127391

[CIT0007] Bidwai PP, Wangoo D, Bhullar NK. 1990 Antispermatogenic action of *Celastrus paniculatus* seed extract in the rat with reversible changes in the liver. J Ethnopharmacol. 28:293–303. 2335957 10.1016/0378-8741(90)90080-d

[CIT0008] Chakraborty J, Singh R, Dutta D, Naskar A, Rajamma U, Mohanakumar KP. 2014 Quercetin improves behavioral deficiencies, restores astrocytes and microglia, and reduces serotonin metabolism in 3-nitropropionic acid-induced rat model of Huntington's disease. CNS Neurosci Ther. 20:10–19. 24188794 10.1111/cns.12189 PMC6493046

[CIT0009] Djousse L, Knowlton B, Cupples LA, Marder K, Shoulson I, Myers RH. 2002 Weight loss in early stage of Huntington's disease. Neurology. 59:1325–1330. 12427878 10.1212/01.wnl.0000031791.10922.cf

[CIT0010] Dong X, Wang Y, Qin Z. 2009 Molecular mechanisms of excitotoxicity and their relevance to pathogenesis of neurodegenerative diseases. Acta Pharmacol Sin. 30:379–387. 19343058 10.1038/aps.2009.24 PMC4002277

[CIT0011] Ellman GL. 1959 Tissue sulfhydryl groups. Arch Biochem Biophys. 82:70–77. 13650640 10.1016/0003-9861(59)90090-6

[CIT0012] Frank S. 2014 Treatment of Huntington's disease. Neurotherapeutics. 11:153–160. 24366610 10.1007/s13311-013-0244-z PMC3899480

[CIT0013] Gattu M, Boss LK, Terry VA, Buccafusco JJ. 1997 Reversal of scopolamine-induced deficits in navigational performance by the seed oil of *Celastrus paniculatus*. Pharmacol Biochem Behav. 57:793–799. 9259008 10.1016/s0091-3057(96)00391-7

[CIT0014] Gil-Mohapel J, Brocardo PS, Christie BR. 2014 The role of oxidative stress in Huntington's disease: are antioxidants good therapeutic candidates? Curr Drug Targets. 15:454–468. 24428525 10.2174/1389450115666140115113734

[CIT0015] Godkar PB, Gordon RK, Ravindran A, Doctor BP. 2003 *Celastrus paniculatus* seed water soluble extracts protect cultured rat forebrain neuronal cells from hydrogen peroxide-induced oxidative injury. Fitoterapia. 74:658–669. 14630170 10.1016/s0367-326x(03)00190-4

[CIT0016] Godkar PB, Gordon RK, Ravindran A, Doctor BP. 2004 *Celastrus paniculatus* seed water soluble extracts protect against glutamate toxicity in neuronal cultures from rat forebrain. J Ethnopharmacol. 93:213–219. 15234755 10.1016/j.jep.2004.03.051

[CIT0017] Godkar PB, Gordon RK, Ravindran A, Doctor BP. 2006 *Celastrus paniculatus* seed oil and organic extracts attenuate hydrogen peroxide- and glutamate-induced injury in embryonic rat forebrain neuronal cells. Phytomedicine. 13:29–36. 16360930 10.1016/j.phymed.2003.11.011

[CIT0018] Gopinath K, Sudhandiran G. 2012 Naringin modulates oxidative stress and inflammation in 3-nitropropionic acid-induced neurodegeneration through the activation of nuclear factor-erythroid 2-related factor-2 signalling pathway. Neuroscience. 227:134–143. 22871521 10.1016/j.neuroscience.2012.07.060

[CIT0019] Gornall AG, Bardawill CJ, David MM. 1949 Determination of serum proteins by means of the biuret reaction. J Biol Chem. 177:751–766. 18110453

[CIT0020] Green LC, Wagner DA, Glogowski J, Skipper PL, Wishnok JS, Tannenbaum SR. 1982 Analysis of nitrate, nitrite, and [^15^N]nitrate in biological fluids. Anal Biochem. 126:131–138. 7181105 10.1016/0003-2697(82)90118-x

[CIT0021] Hertog HJ, Hackman TJ, Nanavati DD, Dev S. 1973 Malkanguniol, one of the polyalcohols from *Celastrus paniculatus* Willd. Tetrahedron Lett. 11:845–848.

[CIT0022] Johri A, Beal MF. 2012 Antioxidants in Huntington's disease. Biochim Biophys Acta. 1822:664–674. 22138129 10.1016/j.bbadis.2011.11.014 PMC3303936

[CIT0023] Kono Y. 1978 Generation of superoxide radical during autoxidation of hydroxylamine and an assay for superoxide dismutase. Arch Biochem Biophys. 186:189–195. 24422 10.1016/0003-9861(78)90479-4

[CIT0024] Krobitsch S, Kazantsev AG. 2011 Huntington's disease: from molecular basis to therapeutic advances. Int J Biochem Cell Biol. 43:20–24. 21056115 10.1016/j.biocel.2010.10.014

[CIT0025] Kumar MHV, Gupta YK. 2002 Antioxidant property of *Celastrus paniculatus* Willd.: a possible mechanism of enhancing cognition. Phytomedicine. 9:302–311. 12120811 10.1078/0944-7113-00136

[CIT0026] Kumar P, Kumar A. 2009a Possible neuroprotective effect of *Withania somnifera* root extract against 3-nitropropionic acid-induced behavioral, biochemical, and mitochondrial dysfunction in an animal model of Huntington's disease. J Med Food. 12:591–600. 19627208 10.1089/jmf.2008.0028

[CIT0027] Kumar P, Kumar A. 2009b Protective effects of epigallocatechin gallate following 3-nitropropionic acid-induced brain damage: possible nitric oxide mechanisms. Psychopharmacology. 207:257–270. 19763544 10.1007/s00213-009-1652-y

[CIT0028] Kumar P, Kumar A. 2010 Protective effect of hesperidin and naringin against 3-nitropropionic acid induced Huntington's like symptoms in rats: possible role of nitric oxide. Behav Brain Res. 206:38–46. 19716383 10.1016/j.bbr.2009.08.028

[CIT0029] Kumar P, Padi SSV, Naidu PS, Kumar A. 2006 Protective effect of antioxidants on 3-nitropropionic acid induced oxidative stress and cognitive impairment. Ann Neurosci. 13:41–47.

[CIT0030] Lekha G, Kumar BP, Rao SN, Arockiasamy I, Mohan K. 2010 Cognitive enhancement and Neuroprotective effect of *Celastrus paniculatus* Willd. seed oil (Jyothismati oil) on male Wistar rats. J Pharm Sci Technol. 2:130–138.

[CIT0031] Luck H. 1971 Catalase In: Bergmeyer HU, editor. Methods of enzymatic analysis. New York: Academic Press; p. 885–893.

[CIT0032] Malik J, Choudhary S, Kumar P. 2015 Protective effect of *Convolvulus pluricaulis* standardized extract and its fractions against 3-nitropropionic acid-induced neurotoxicity in rats. Pharm Biol. 53:1448–1457. 25853968 10.3109/13880209.2014.984856

[CIT0033] Mittal SK, Eddy C. 2013 The role of dopamine and glutamate modulation in Huntington disease. Behav Neurol. 26:255–263. 22713410 10.3233/BEN-2012-120268 PMC5215439

[CIT0034] Nadkarni KM. 2002 Indian Materia Medica. New Delhi: Popular Prakashan Pvt. Ltd.

[CIT0035] Nalini K, Karnath KS, Rao A, Aroor AR. 1995 Effects of *Celastrus paniculatus* on passive avoidance performance and biogenic amine turnover in albino rats. J Ethnopharmacol. 47:101–108. 7500635 10.1016/0378-8741(95)01264-e

[CIT0036] Palmer GC. 2001 Neuroprotection by NMDA receptor antagonists in a variety of neuropathologies. Curr Drug Targets. 2:241–271. 11554551 10.2174/1389450013348335

[CIT0037] Perez-De La Cruz V, Elinos-Calderon D, Robledo-Arratia Y, Medina-Campos ON, Pedraza-Chaverri J, Ali SF, Santamaria A. 2009 Targeting oxidative/nitrergic stress ameliorates motor impairment, and attenuates synaptic mitochondrial dysfunction and lipid peroxidation in two models of Huntington's disease. Behav Brain Res. 199:210–217. 19100293 10.1016/j.bbr.2008.11.037

[CIT0038] Picconi B, Passino E, Sgobio C, Bonsi P, Barone I, Ghiglieri V, Pisani A, Bernardi G, Ammassari-Teule M, Calabresi P. 2006 Plastic and behavioral abnormalities in experimental Huntington's disease: a crucial role for cholinergic interneurons. Neurobiol Dis. 22:143–152. 16326108 10.1016/j.nbd.2005.10.009

[CIT0039] Rajkumar R, Kumar EP, Sudha S, Suresh B. 2007 Evaluation of anxiolytic potential of *Celastrus* oil in rat models of behaviour. Fitoterapia. 78:120–124. 17169502 10.1016/j.fitote.2006.09.028

[CIT0040] Saydoff JA, Liu LS, Garcia RA, Hu Z, Li D, von Borstel RW. 2003 Oral uridine pro-drug PN401 decreases neurodegeneration, behavioral impairment, weight loss and mortality in the 3-nitropropionic acid mitochondrial toxin model of Huntington's disease. Brain Res. 994:44–54. 14642447 10.1016/j.brainres.2003.09.049

[CIT0041] Shinomol GK, Muralidhara 2011 *Bacopa monnieri* modulates endogenous cytoplasmic and mitochondrial oxidative markers in prepubertal mice brain. Phytomedicine. 18:317–326. 20850955 10.1016/j.phymed.2010.08.005

[CIT0042] Silva RH, Abilio VC, Kameda SR, Takatsu-Coleman AL, Carvalho RC, Ribeiro Rde A, Tufik S, Frussa-Filho R. 2007 Effects of 3-nitropropionic acid administration on memory and hippocampal lipid peroxidation in sleep-deprived mice. Prog Neuropsychopharmacol Biol Psychiatry. 31:65–70. 16876303 10.1016/j.pnpbp.2006.06.019

[CIT0043] Sugino T, Nozaki K, Takagi Y, Hattori I, Hashimoto N, Yodoi J. 1999 Expression and distribution of redox regulatory protein, thioredoxin after metabolic impairment by 3-nitropropionic acid in rat brain. Neurosci Lett. 275:145–148. 10568520 10.1016/s0304-3940(99)00763-6

[CIT0044] Tandon N, Sharma P. 2011 Quality standards of Indian medicinal plants. New Delhi: Medicinal Plants Unit, Indian Council of Medical Research.

[CIT0045] Tu YQ, Chen YZ. 1993 Sesquiterpenoids from *Celastrus paniculatus*. J Nat Prod. 56:122–125.

[CIT0046] Tu YQ, Wu TX, Li ZZ, Zhen T, Chen YZ. 1991 Sesquiterpene polyol esters from *Celastrus paniculatus*. J Nat Prod. 54:1383–1386.

[CIT0047] Tunez I, Santamaria A. 2009 Model of Huntington's disease induced with 3-nitropropionic acid. Rev Neurol. 48:430–434. 19340784

[CIT0048] Tunez I, Tasset I, Cruz VPL, Santamaria A. 2010 3-Nitropropionic acid as a tool to study the mechanisms involved in Huntington's disease: past, present and future. Molecules. 15:878–916. 20335954 10.3390/molecules15020878 PMC6263191

[CIT0049] Wagner H, Heckkel E, Sonnenbichler J. 1974 Isolation and structures of a new sesquiterpene (malkangunin) and two new sesquiterpene alkaloids (celapanin, celapanigin) from *Celastrus paniculatus* Willd. Tetrahedron Lett. 2:213–216.

[CIT0050] Wills ED. 1966 Mechanisms of lipid peroxide formation in animal tissues. Biochem J. 99:667–676. 5964963 10.1042/bj0990667 PMC1265056

